# Supplementary material for: Genome-Wide Identification and Analysis of the Genes Encoding Q-Type C2H2 Zinc Finger Proteins in Grapevine
Source: Int J Mol Sci. 2023 Oct 14;24(20):15180. doi: 10.3390/ijms242015180 (PMC10607507; doi:10.3390/ijms242015180)
Supplement: Supplementary file 1 [file ijms-24-15180-s001.zip › Caption of Supplementary Figures and Tables.pdf]

Supplementary Figure S1. Relative synonymous codon usage (RSCU) of *VvZFP* genes. Note: Codon RSCU values were performed using the web-based program CodonW 1.4.2. A heat map of RSCU values of the *VvZFP* genes was drawn using TBtools 1.118.0.0. The color from aqua to violate represents the RSCU value from low to high.

Supplementary Figure S2. Relative synonymous codon usage (RSCU) of *VrZFP* genes. Note: Codon RSCU values were performed using the web-based program CodonW 1.4.2. A heat map of RSCU values of the *VrZFP* genes was drawn using TBtools 1.118.0.0. The color from aqua to violate represents the RSCU value from low to high.

Supplementary Figure S3. Relative synonymous codon usage (RSCU) of *VaZFP* genes. Note: Codon RSCU values were performed using the web-based program CodonW 1.4.2. A heat map of RSCU values of the *VaZFP* genes was drawn using TBtools 1.118.0.0. The color from aqua to violate represents the RSCU value from low to high.

Supplementary Table S1. Oligo Sequence of *VvZFP* family gene.

Supplementary Table S2. Cis-acting elements of *VvZFP*, *VrZFP*, and *VaZFP.*

Supplementary Table S3. Parameters for quantification of codon usage preference for VvZFP, VaZFP, and VrZFP codons.

Supplementary Table S4. Ka/Ks analysis of *VvZFP*, *VaZFP*, and *VrZFP.*

Supplementary Table S5. The interspecies collinearity analysis between the *VvZFPs* and the other five species.

Supplementary Table S6. The expression levels in different tissues of *VvZFPs.*
